# Three-phase alternating current liquid metal vortex magnetohydrodynamic generator

**DOI:** 10.1016/j.isci.2021.102644

**Published:** 2021-05-24

**Authors:** Siddharth Raj Gupta, J. Ashley Taylor, Tom Krupenkin

**Affiliations:** 1 University of Wisconsin-Madison, Department of Mechanical Engineering, Madison, WI 53706, USA; 2 University of Wisconsin-Madison, Department of Electrical and Computer Engineering, Madison, WI 53706, USA

**Keywords:** physics, electricity, engineering, energy engineering, mechanical engineering, electrical property

## Abstract

Magnetohydrodynamic (MHD) generators directly convert mechanical energy to electrical energy. However, due to production of low amplitude voltages at low fluid velocities, they are not useful for electronic devices requiring power at watt scale. This work introduces vortex MHD, capable of producing voltages on scale of volts and generating power on a scale of watts. This is achieved by using Galinstan, a highly conductive metallic fluid, which remains liquid at room temperature. The proposed device comprises an impeller and set of copper coils positioned in a ferromagnetic housing. Three-phase AC current is passed in the coils producing a rotating magnetic field. The interaction of a moving conductive fluid and rotating magnetic field governed by Faraday's law of induction serves as a mechanism of electrical current generation. The study investigates the system performance and, in particular, variation of power with respect to system parameters like fluid inlet velocity and stator current.

## Highlights

• A novel liquid metal vortex MHD generator with Watt scale power output is proposed
• AC voltage on a scale of Volts can be produced without any transformer
• Variation of output power with fluid velocity, rotor RPM, and slip are investigated

## Graphical abstract

**Figure:**
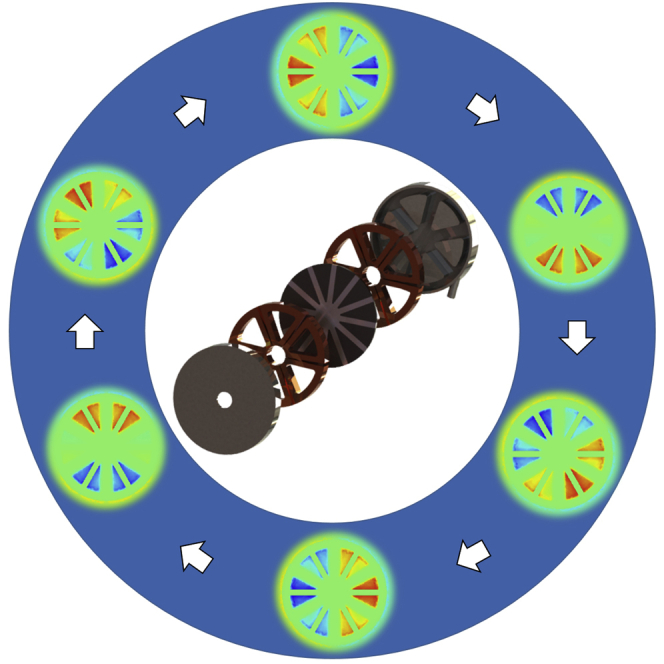

## Introduction

Recent developments in technology have dramatically increased our dependency on mobile electronics and Internet of Things devices (Kang et al., 2019; Caro and Sadr, 2019). Most of these devices either run on batteries or require a permanent connection to the power grid, which restricts their use in many applications. The growing demand for wearable (Bartlett et al., 2016; Miner et al., 2001) and portable devices (Widdicks et al., 2017; Ghose and Han, 2014) is stimulating a further investigation of various alternative sources of energy. Lithium-ion batteries are a common solution, but they have a limited life span and require regular recharging. Various energy harvesting technologies which show a potential to produce power in the range of μW to mW have been investigated in the past. Some common examples include piezoelectricity (Muralt et al., 2009), as well as electrostatic (Roundy et al., 2003) and triboelectric (Fan et al., 2012) energy generation. The recently developed method of reverse electrowetting (Hsu et al., 2015) also demonstrated the ability to produce power on the scale of watts. While traditional mechanical systems have multiple moving solid parts, a liquid-based energy harvesting approach is often preferable because of its ability to convert high pressure to high velocities using a simple converging nozzle. Such fluid-based systems are much more advantageous in harvesting energy from mechanical energy sources characterized by high forces and low displacements.

Magnetohydrodynamic (MHD) generators are a class of devices, which can directly convert the mechanical energy of the flowing fluid into electrical energy. MHDs can be separated into two broad groups: (i) direct current (DC) MHDs, (ii) alternating current (AC) MHDs, depending on the type of output produced. Michael Faraday in 1832 first demonstrated DC MHD generators, which required Ohmic contact with the conductive fluid for the flow of current (Strohl and Jackson, 2021). This discovery led to a plethora of studies in MHD technology being conducted in later parts of the 19^th^ and 20^th^ centuries. Woodson, H. H in 1962 proposed various kinds of AC MHD designs (Woodson, 1964). Bernstein et al. laid the foundation of electrodeless MHD (Bernstein et al., 1961), and Jackson et al. performed various studies on MHD induction generators in 1964 (Jackson and Pierson, 1964). With the growing energy demand, MHD has gained a lot of attention in recent years because of the simple design and direct conversion of mechanical to electrical energy. Pattana et al. in 2010 analyzed the performance of a radial flow disc-based MHD using the finite element method end confirmed the possibility to produce an AC power with a single side excitation (Intani et al., 2010). Panchadar et al. demonstrated a vortex flow DC generator capable of producing 34 W/cm^3^ of power density (Panchadar et al., 2019). However, similar to other DC MHDs, it had a limitation of generating low voltages at low flow velocities. To increase the output voltage, West et al. demonstrated a novel vortex flow generator integrated with a liquid switch and an electrical transformer (West et al., 2020).

**Figure 1:**
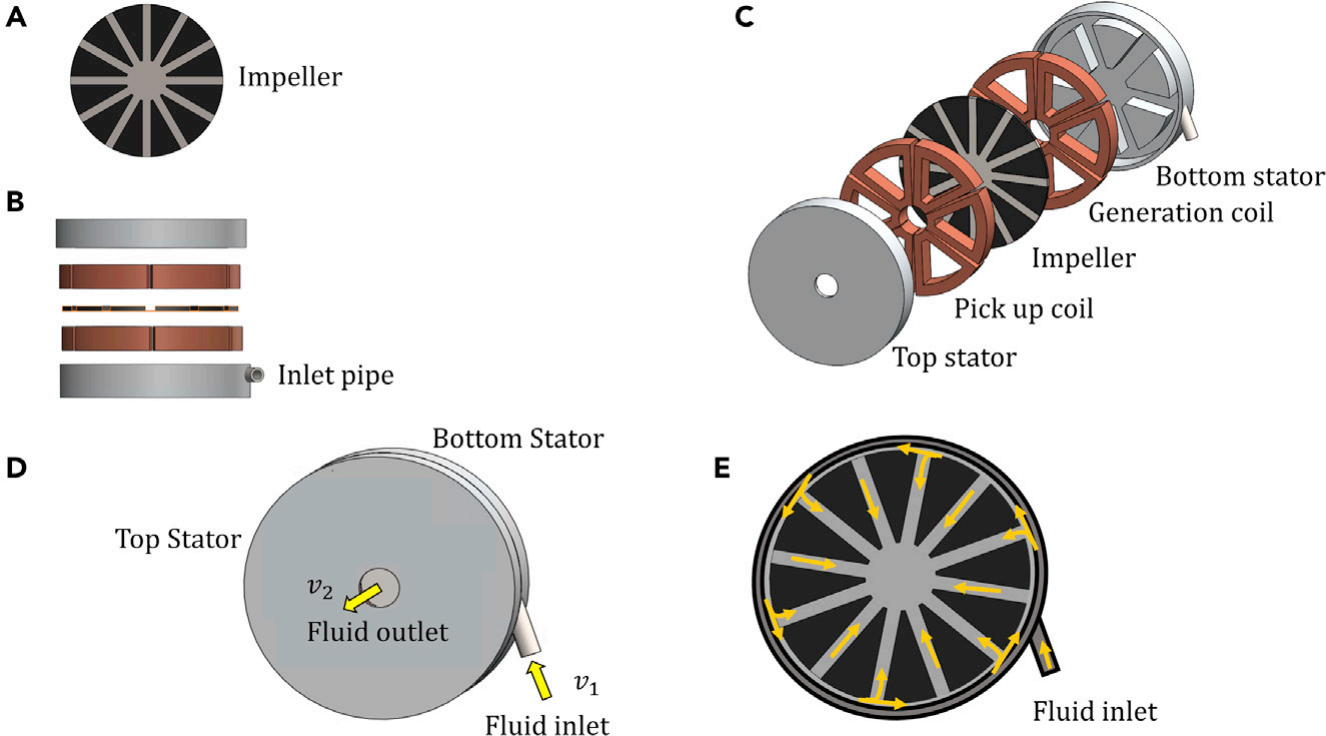
Model setup (A) Geometry of the ferromagnetic impeller. (B) Geometry of the side view of the generator. (C) Geometry of the overall model assembly. (D) Direction of fluid flow with inlet and outlet of fluid. (E) Cross-sectional schematic.

Limitations of DC MHD generators make it difficult to use them at low fluid flow velocities. Even though the previously studied vortex designs allow high power density and resolve the issue of end losses, they still fail to address the problems regarding the low output voltage. To overcome these limitations, the present study investigates a novel vortex based MHD device, which works similar to an asynchronous generator capable of producing AC voltages. The proposed device is free of any Ohmic contact with the flowing fluid and does not require any additional switch or transformer. To obtain a higher value of the magnetic field in the rotor domain, a double stator arrangement as shown in Figure 1 is considered. The behavior of the proposed device has certain similarities to the traditional asynchronous generators, which allow us to utilize various analogies with the classical induction generator theories.

## Results

### Model setup

Fluid-based systems have an advantage of having a few moving parts in comparison to traditional mechanical generators, which often have complex geometries and a number of solid parts moving relative to each other. Direct conversion of mechanical energy of the flowing fluid into electrical energy can greatly reduce the complexity of the generator system providing substantial benefits over traditional generators. Moreover, using highly conductive liquid metals like Mercury or Galinstan ensures low internal resistances and provides a possibility of generating high power even at low fluid velocities.

Previously conducted MHD studies utilized a linear duct flow of the fluid, but those designs are prone to various disadvantages (Wang and Dudzinsky, 1967a, 1967b). Linear duct flow suffers substantial end losses due to the edge effects, whereas the proposed MHD design incorporates vortex motion of the fluid in a cylindrical geometry removing the possibility of end losses.

The physical structure of the MHD generator under consideration is presented in Figure 1. The setup comprises a pair of three-phase coil windings positioned in a ferromagnetic housing along with a ferromagnetic iron impeller disposed in between the two coils. Upon injection through the pipe inlet, liquid—metal travels circumferentially and radially to reach the center as shown in Figures 1D and 1E. The fluid entering through the inlet pipe creates large dynamic pressure, which causes the impeller to rotate around its axis. An ideal design of the generator would be to have just the vortex flow of the fluid in between two stators without any impeller. However, the presence of an impeller provides several advantages in the proposed setup. Firstly, the role of the impeller is not to produce a torque but to create a well-defined path for directing the flow of the metallic conducting fluid. Secondly, because of the high relative permeability of iron ($\mu_{r}$= 5,000), the presence of an impeller reduces the magnetic reluctance of the circuit, thereby allowing the system to achieve a higher magnetic field with an average value of approximately 1 Tesla.

Some of the possible applications of the proposed device include small-scale electronic devices requiring power on a Watt scale. Therefore, an effort has been made to keep the device's form-factor as compact as possible. In view of this, the considered device is selected to have an outer diameter of 48 mm and a thickness of 5 mm for each stator and 1 mm thickness for the impeller.

### Device operation

The model is set up as a volumetric flow driven device. Hence, the fluid enters the generator through the inlet at a constant velocity as shown in Figure 1D. Since the impeller is rotating, the motion of each element of fluid is a vector sum of radial and tangential flow as schematically shown in Figure 1E. The fluid travels in circumferential and radial directions reaching the center and exiting perpendicular to the plane of the stator with a velocity v_2_. Since the considered velocities are well below the speed of sound, the incompressible fluid continuity equation relates the inlet and the outlet velocities. With the outlet area much larger than the inlet area, v_2_ is expected to be smaller than v_1_ as expressed by Equation 1.

(Equation 1) $$A_{1}\;v_{1}=\;A_{2}\;v_{2}\;$$

**Figure 2:**
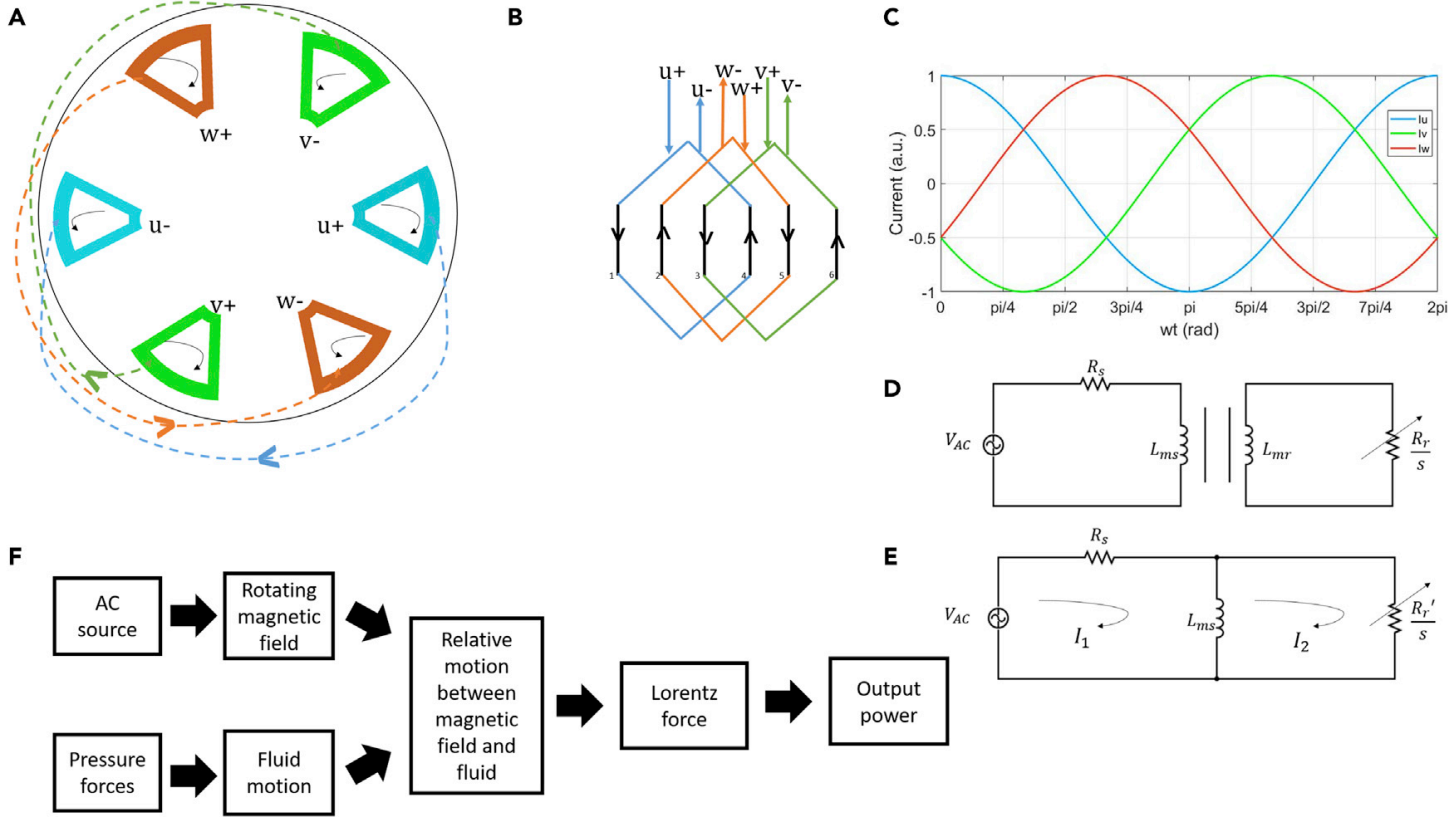
Electrical overview and schematic workflow (A) Actual coil connections. (B) Coil winding diagram. (C) Three-phase current variation. (D) Per-phase exact equivalent circuit. (E) Per-phase approximated equivalent circuit. (F) Schematic describing the working of generation operation.

The device has two sets of copper coil windings that are responsible for the creation of a three-phase rotating magnetic field similar to a standard AC asynchronous induction generator (Lipo, 2017). Figures 2A and 2B describe the coil winding structure and winding connections. The three-phases of the coils are marked as u, v, and w and are wound over the ferromagnetic mounts present in the stator as shown in Figure 1C. The positive and the negative terminals of the same phase are 180° apart, which corresponds to the full pitch two pole single layer asynchronous generator layout (Lipo, 2017). To generate the rotating field, the currents in the three phases keep a 120° phase difference between each other and take the format governed by Equations 2, 3, and 4 as represented in Figure 2C.

(Equation 2) $$I_{u}=I_{0}\;\cos\left(wt\right)$$

(Equation 3) $$I_{v}=I_{0}\;\cos\left(wt+\frac{2\pi }{3}\right)\;$$

(Equation 4) $$I_{w}=I_{0}\;\cos\left(wt+\frac{4\pi }{3}\right)$$

where $I_{0}$ is the peak value of the supplied current.

**Figure 3:**
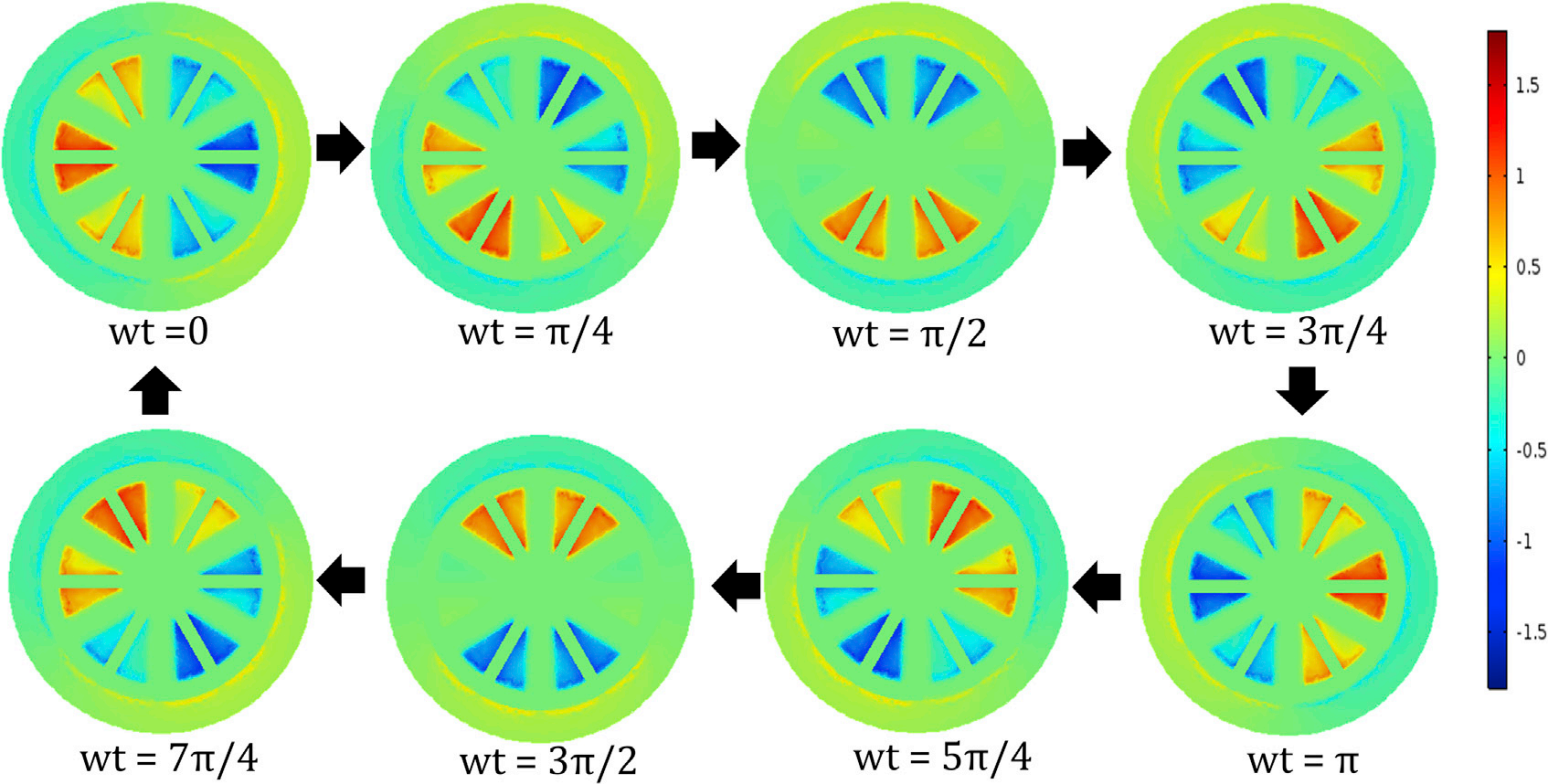
Rotating magnetic field

Figure 3 shows the magnetic field and its variation with time obtained with the help of the Finite Element Method (FEM). These FEM calculations were performed based on the B-H saturation curve given in the supplemental information (Figure S2). As can be seen from Figure 3, the magnetic field starts to saturate at the edges with a value around 1.5 T, which is consistent with the B-H curve. The peak value of the magnetic field was achieved by using equal amplitude three-phase currents corresponding to $I_{0}$ = 400 mA in each phase of the generation coil having 50 turns. The FEM simulations further show that such a setup produces an average magnetic field of 0.81 T. Further details of the model including the size of the wires, type of material, etc. are presented in the supplemental information.

The current is induced in liquid metal in the same way as it is induced in the squirrel cage (Lipo, 2017) of an induction generator. This induced current influences the operating current in top and bottom stator coil windings. Conceptually, both the top and bottom windings can be shared for the generation and extraction of the output voltage. However, it is simpler to use one of the coil windings as the generation coil and the other as a pick-up coil. In the model described in Figure 1, the bottom stator coil acts as a generation coil and the top stator coil acts as a pick-up or extraction coil.

### Working principle

The three-phase current as shown in Figure 2C produces a magnetic field (Figure 3) that rotates in time due to the sinusoidal behavior of the governing AC currents. Synchronous machines rotate the field at the same speed as of rotor however, an asynchronous machine incorporates a difference in the running speed. This relative difference in speed called slip (also known as slip ratio) (Lipo, 2017) is calculated using Equation 5.

(Equation 5) $$s=\;\frac{w_{s}-\;w_{r}}{w_{s}}$$

where $w_{s}$ is synchronous speed and $w_{r}$ is the speed of the rotor.

For an induction machine to work as a generator, the rotational speed of the rotor should be greater than the synchronous speed, which corresponds to the slip having negative values. This relative speed along with the rotating magnetic field induces the current in the rotor in a similar way it induces a current in the bars of the squirrel cage of the induction generator (Lipo, 2017). To comply with Lenz's law, these induced rotor currents thereby produce a magnetic field, which influences the current in the stator.

The block diagram representation of the device operation is shown in Figure 2F. It can be seen that the pressure forces provided by the external sources, accelerate the liquid into the fluid inlet, which thus rotates the impeller because of the dynamic pressure of the fluid. The combination of the relative fluid motion and the supplied magnetic field leads to the production of induced voltage, which is given by the cross product of velocity and the total perpendicular magnetic field. This induced AC current is thereby the result of the Lorentz force experienced by the charge carriers in the fluid, which in turn generates the output power.

The exact and the approximated equivalent circuit of the model under consideration are represented in Figures 2D and 2E. The model is similar in function to a transformer with $R_{s}$ and $L_{ms}$ being the stator resistance and inductance, and with $V_{AC}$ being the applied voltage to the stator, which produces a stator magnetic field. These parameters are calculated using FEM analysis (Table S2 and Table S3) and the values are presented in the Table S2. The approximate equivalent circuit parameters indicated by primed variables in Figure 2E are obtained by multiplying the original parameters with an effective turn ratio $a_{eff}\;$as shown in Equation 6, where $a_{eff}\;$ is the ratio of self-inductances of the stator and rotor.

(Equation 6) $$R_{r}^{\prime }=\;a_{eff}\;R_{r}$$

The equations used to obtain the approximate circuit variables along with the derivation of primed variables are presented in the supplemental information. As seen in Figures 1D and 1E the fluid enters through the nozzle and hit the impeller surface, therefore, for calculation of rotor resistance$\left(R_{r}\right)$, the path between the two-impeller blades is taken as equivalent to the rotor coil wounded over the iron impeller.

## Discussion

The system parameter values are obtained using the finite element method and analytical calculations, with the details given in the supplemental information. After knowing the required parameter values, Kirchhoff's current and voltage laws are used to solve the equivalent circuit represented in Figures 2D and 2E. With $I_{1}$ and $I_{2}$ taken as the Kirchhoff's loop currents, the circuit calculations are shown in the supplemental information.

The power transmitted from stator field to the rotor ($P_{in}$) is calculated using Equations S32 to S35 given in the supplemental information and is summarized in Equation 7.

(Equation 7) $$P_{in}=3\;|I_{2}{|}^{2}\;\frac{{R_{r}}^{'}}{s}$$

Positive slip implies positive power with the rotor speed less than synchronous speed indicating motor operations. Whereas negative slip means rotor speed is higher than the synchronous speed and the machine is acting as a generator. The power dissipated in the rotor is then calculated using Equation 8.

(Equation 8) $$P_{diss}=\;P_{in}\;s$$

Therefore, the difference between the power transmitted from stator field to the rotor and power dissipated in the rotor gives the converted power, as shown by Equation 9.

(Equation 9) $$P_{con}=\;P_{in}\;-\;P_{diss}$$

To investigate the effect of slip on the output power, a case with magnetic flux of 5∗10^−5^ Wb was studied. FEM calculations showed that this could be achieved by using 0.4 A current in 50 turns stator coil, which produces an average magnetic field of 0.7 T. The detailed calculations of the average magnetic field are given in the supplemental information. Further using Kirchhoff's law analysis it was established that such a system requires a supply voltage with the peak values of approximately 0.96 V.

**Figure 4:**
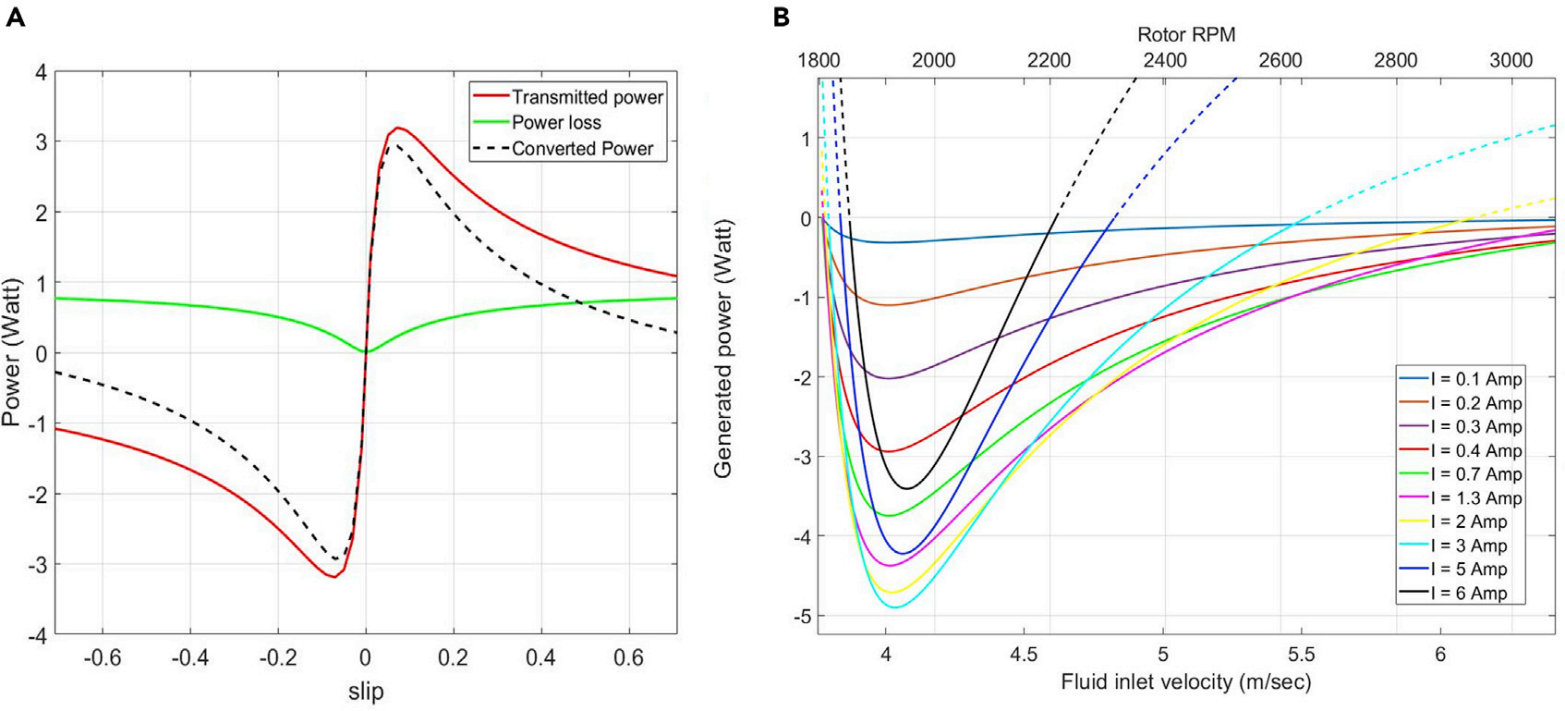
Power profile (A) Variation of power transmitted from stator field to the rotor, power loss, and converted power with the slip. (B) Variation of converted power with rotor RPM, fluid inlet velocity, and applied current.

Figure 4A shows the variation of power with respect to slip. Positive power corresponds to the motor operation where the power is consumed whereas negative power corresponds to the generator operation where the power is produced. It can be seen that the device is capable of producing powers up to 3 W when the applied current is 0.4 A. The converted (output) power is zero at zero slip, which corresponds to the synchronous operation when there is no relative motion between the stator field and the rotor. Figure 4B shows that the converted power increases with an increase in the absolute value of slip and attains maximum, after which the rotor losses become more dominant and the power starts to decrease.

The rotor speed can be written in terms of slip (Lipo, 2017) as shown in Equation 10.

(Equation 10) $$w_{r}=w_{s}\;\left(1-s\right)$$

In the model considered, the stator has 6 coils for 3 phases, which corresponds to 2 poles (or 1 pole pair), whereas the rotor has 12 impeller fins that act as rotor poles. Therefore, the model consists of total 6 rotor pole pairs or 2 rotor pole pairs per phase of the stator, which are double the number of stator pole pairs.

(Equation 11) $$w_{r}^{\left(electrical\right)}=p\;w_{r}^{\left(mechanical\right)}$$

Equation 11 represents the relation between the electrical and mechanical speed of the rotor, where p is the number of pole pairs (p = 2) in the model considered.

Further, Equation 12 relates the mechanical speed of the rotor with the fluid injection velocity $v_{1}$ and outer radius r, which is equal to 24 mm.

(Equation 12) $$w_{r}^{\left(mechanical\right)}=\frac{v_{1}}{r}$$

Hence using Equations 10, 11, and 12, a new relation is derived in Equation 13, which relates the fluid inlet velocity and the slip value.

(Equation 13) $$v_{1}=w_{s}\;r\frac{1-s}{p}\;$$

In Figure 4B, the variation of output power with the rotor RPM and fluid inlet velocity is shown as a function of the supplied current. It can be seen that the produced power increases as we increase the current from 0.1 A to 3 A but starts to decrease as we move to 6 A. This behavior is due to two reasons. First, the saturation effect of the material does not let the magnetic field increase beyond its saturation limit, and second, an increase in current increases the loss due to Joule heating. The dotted curves in Figure 4B represents the fluid velocities or the rotor RPMs where the losses are dominant and the generator consumes power instead of producing it. At very small inlet velocities, Joule heating governs the generator's power and hence lower current produces higher power initially. However, as velocity increases, rotor losses become more prominent due to an increase in the rotor RPM, and the generator produces lower power even at a higher current for the same velocity. This can be seen in Figure 4B where for the fluid velocity of 5.5 m/s, the power generated at 0.4 A is greater than the power generated at 2 A.

**Figure 5:**
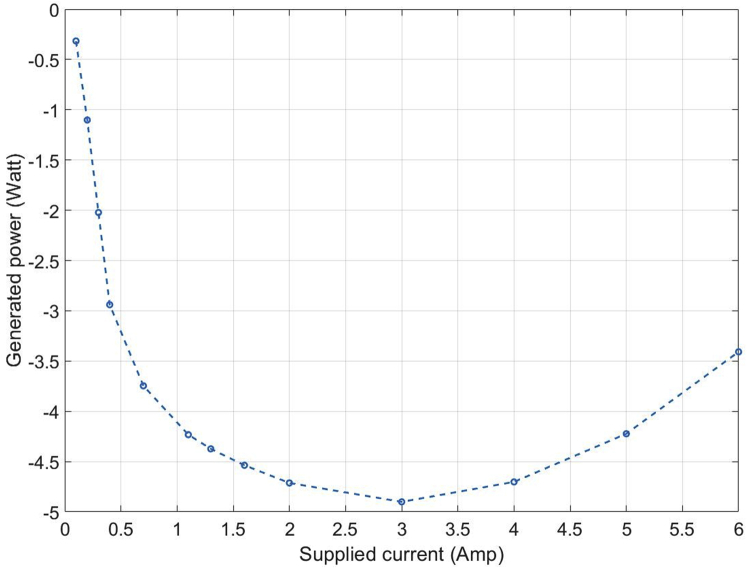
Peak power as a function of supplied current

Figure 5 shows the variation of the peak power (corresponding to the minima of the curve in Figure 4B) with the supplied current. Table 1 shows that the magnetic field starts to saturate around 0.7 A, and therefore, it can be seen from Figure 5 that while the generator is operating in a linear magnetic region, the power varies as the square of magnetic field corresponding to the supplied current. Further increase in current drives the system to saturation and the generated power starts to decrease after peaking at 3 A. This is because the higher currents create extensive resistance losses, which start to dominate as the input current is further increased. The rotor RPM corresponding to the different power peaks in Figure 5 varies between the range of 1900 RPM to 2000 RPM and the specific values of rotor RPM and fluid inlet velocity corresponding to these peaks can be obtained using Figure 4B, Equations 10 and 13.

**Table 1:** Magnetic field as a function of supplied current

**Current (A)**	0.1	0.2	0.3	0.4	0.7	1.1	1.3	1.6	2	3	4	5	6
**B (T)**	0.23	0.43	0.58	0.70	0.80	0.85	0.87	0.89	0.93	0.99	1.05	1.10	1.14

Our proposed device can be used to power various Internet of Things (IoT) devices. One such area is high force (between 1000N and 10000N) and low displacement (between 1mm–20mm) operation. A possible application is in transportation using shipping containers where the continuous power output from MHD can help provide real time positioning of the containers. This has been discussed in detail in the supplemental information.

### Conclusion

The paper presents a concept of novel vortex magnetohydrodynamic (MHD) generator free from end losses and capable of producing AC voltages on a scale of volts and output power on a scale of watts. The proposed device overcomes common problems of DC MHDs including low output voltages at low fluid velocities and operates on a principle similar to an asynchronous induction generator. The present study investigates the dependence of the output power on applied current, fluid injection velocity, and the slip value. A combination of analytical theory and FEA simulations was used to obtain the optimal values of the system parameters such as inductances, resistances, and the rotor velocity. The proposed device has a simple geometry, which improves its reliability and allows effective coupling to a large number of mechanical energy sources. It was demonstrated that the device is capable of producing up to 3 W of power; however, it can be optimized to further increase the output power. To achieve this, a more conductive metallic fluid can be used to increase the output power without introducing any conceptual modifications.

## STAR★methods

### Key resources table

**Table:** 

REAGENT or RESOURCE	SOURCE	IDENTIFIER
**Software and algorithms**		

COMSOL	COMSOL Inc.	https://www.comsol.com/
MATLAB	MathWorks	https://www.mathworks.com/
SOLIDWORKS	Dassault Systems	https://www.solidworks.com/

### Resource availability

#### Lead contact

Further information and requests for resources and material should be directed to and will be fulfilled by the lead contact, Tom Krupenkin (tnk@engr.wisc.edu).

#### Material availability

This study did not generate new unique materials.

#### Data and code availability

This study did not analyze any pre-existing code, however, the data generated by Finite Element Method is listed in the supplemental information (Table S2).

### Method details

#### Material specifications

To maximize the linear magnetic region, it was required to select a material, which saturates at higher values of current. For this purpose, the proposed model uses easily available Silicon steel NGO 35JN200 for both the stators and impeller. Each stator ferromagnetic mount on which the coil winds is 5 mm in thickness and since the number of turns per coil are taken to be 50, the thickness of each wire equals 0.1 mm which corresponds to № 38 AWG wire**.**

#### Computational and Finite Element Method modeling

Computational experiments and numerical calculations were performed using the Finite Element Method (FEM). The per-phase approximated equivalent circuit (Figure 2E) is first evaluated computationally using Kirchhoff’s law to obtain the values of system parameters on the rotor side of the circuit. The detailed calculations and derivations are presented in the supplemental information. To investigate the variation of power with respect to applied current and fluid inlet velocity, 10 different cases were studied (Table S1 and Table S2). To demonstrate this, the applied current is varied from 0.1 A to 6 A to establish the current regimes where power increases with an increase in current and then decreases with further increase in current. This can be seen in Figure 5. For the purpose of FEM modeling, silicon steel was considered to ensure the availability of a larger linear magnetic region, and the number of turns in each copper coil was taken to be 50. Further material and modeling details, parameter values, calculations of magnetic flux, and average magnetic field in the system along with the governing equations are presented in the supplemental information.

##### Equations related to circuit

The equivalent circuit along with the method to calculate the referred variables are based on the following equations presented below.

Consider a general case of the induction machine, with $I_{s}$ and $I_{r}$ as current, $L_{ms}$ and $L_{mr}$ as self-inductance and $L_{sr}$ and $L_{rs}$ as mutual inductances for stator and rotor respectively.

The total amount of flux linkages in the stator and rotor coil are written as

(Equation S1) $$\begin{array}{l} \Lambda_{s}=L_{ls}\;\times I_{s}+L_{ms}\;\times I_{s}+\;L_{sr}\;\times I_{r}\; \end{array}$$

(Equation S2) $$\begin{array}{l} \Lambda_{r}=L_{lr}\;\times I_{r}+L_{mr}\;\times I_{r}+\;L_{rs}\;\times I_{s} \end{array}$$

Rearranging terms in Equation S1

(Equation S3) $$\begin{array}{l} \Lambda_{s}=L_{ls}\;\times I_{s}+L_{ms}\;\left(I_{s}+\;\frac{L_{sr}}{L_{ms}}\;\times I_{r}\right) \end{array}$$

Defining a new parameter

(Equation S4) $$\begin{array}{l} I_{r}^{\prime }=\;\frac{L_{sr}}{L_{ms}}\;\times I_{r}\; \end{array}$$

Therefore

(Equation S5) $$\begin{array}{l} I_{r}=\;\frac{L_{ms}}{L_{sr}}\;\times I_{r}^{\prime }\; \end{array}$$

Using Equations S4 in S3

(Equation S6) $$\begin{array}{l} \Lambda_{s}=L_{ls}\;\times I_{s}+L_{ms}\;\times \left(I_{s}+\;I_{r}^{\prime }\right) \end{array}$$

Using Equations S5 in S2

(Equation S7) $$\begin{array}{l} \Lambda_{r}=L_{lr}\;\times \frac{L_{ms}}{L_{sr}}\;\times I_{r}^{\prime }+L_{mr}\;\times \;\frac{L_{ms}}{L_{sr}}\;\times I_{r}^{\prime }+\;L_{rs}\;\times I_{s}\; \end{array}$$

Rearranging Equation S7

(Equation S8) $$\begin{array}{l} \Lambda_{r}=L_{lr}\;\times \frac{L_{ms}}{L_{sr}}\;\times I_{r}^{\prime }+L_{rs}\;\times \left(\;I_{s}+\;\frac{L_{mr}}{L_{rs}}\;\times \;\frac{L_{ms}}{L_{sr}}\;\times I_{r}^{\prime }\right) \end{array}$$

Multiply Equation S8 both sides by $\;\frac{L_{ms}}{L_{rs}}\;$

(Equation S9) $$\begin{array}{l} \Lambda_{r}\times \;\frac{L_{ms}}{L_{rs}}=L_{lr}\;\times \frac{L_{ms}^{2}}{L_{sr}\times \;L_{rs}}\;\times I_{r}^{\prime }+L_{ms}\;\times \left(\;I_{s}+\;\frac{L_{mr}}{L_{rs}}\;\times \;\frac{L_{ms}}{L_{sr}}\;\times I_{r}^{\prime }\right) \end{array}$$

Introducing a new variable $\Lambda_{r}^{\prime }=\;\Lambda_{r}\times \;\frac{L_{ms}}{L_{rs}}$ and $\;L_{lr}^{\prime }=\;L_{lr}\times \frac{L_{ms}^{2}}{L_{sr}\times \;L_{rs}}$. Therefore Equation S9 can be written as

(Equation S10) $$\begin{array}{l} \Lambda_{r}^{\prime }=L_{lr}^{\prime }\times I_{r}^{\prime }+L_{ms}\;\times \left(\;I_{s}+\;\frac{L_{mr}}{L_{rs}}\;\times \;\frac{L_{ms}}{L_{sr}}\;\times I_{r}^{\prime }\right) \end{array}$$

The primed variables are interpreted as equivalent quantities referred to the stator turns by effective turns ratios $a_{eff}\;$, which is given by Equation S11

(Equation S11) $$\begin{array}{l} a_{eff}=\;{\left(\frac{N_{1}}{N_{2}}\right)}^{2}{\left(\frac{p_{2}}{p_{1}}\right)}^{2}{\left(\frac{p_{3}}{N_{3}}\right)}^{2} \end{array}$$

where N_1_, N_2,_ and N_3_ are the number of turns and p_1_, p_2_ and p_3_ are the number of poles of the bottom stator, top stator, and rotor respectively. The values of N_1_ and N_2_ are taken to be 50 while N_3_ is taken to be 1 as the rotor can be considered as a short-circuited winding. The number of pole pairs p_1_, p_2_ in both the stator windings are considered to be 1 while the rotor is considered to have 2 pole pairs. The voltage equation across the rotor can be written as shown in Equation S12.

(Equation S12) $$\begin{array}{l} V_{r}=\;I_{r}\times \;R_{r}+\frac{d}{dt}\;\Lambda_{r}\; \end{array}$$

Equation S12 can be transformed into stator turns by multiplying it both sides by $\;\frac{L_{ms}}{L_{rs}}\;$

(Equation S13) $$\begin{array}{l} V_{r}\;\times \;\frac{L_{ms}}{L_{rs}}=\frac{L_{ms}}{L_{rs}}\times \;I_{r}\times \;R_{r}+\frac{L_{ms}}{L_{rs}}\times \frac{d}{dt}\;\Lambda_{r} \end{array}$$

Introducing new variables $V_{r}^{\prime }\;$ such that

(Equation S14) $$\begin{array}{l} V_{r}^{\prime }=\;V_{r}\;\times \;\frac{L_{ms}}{L_{rs}}\; \end{array}$$

Using $\Lambda_{r}^{\prime }=\;\frac{L_{ms}}{L_{rs}}\times \Lambda_{r}$ , Equations S5 and S14, in Equation S13, we can obtain Equation S15

(Equation S15) $$\begin{array}{l} V_{r}^{\prime }=\;I_{r}^{\prime }\times \;R_{r}^{\prime }+\frac{d}{dt}\Lambda_{r}^{\prime }\; \end{array}$$

where

(Equation S16) $$\begin{array}{l} R_{r}^{\prime }=\frac{L_{ms}^{2}}{L_{sr}\times L_{rs}}\;\times \;R_{r}\; \end{array}$$

Since mutual inductance can be reciprocated we can use $L_{sr}=\;L_{rs}$. Now, considering an ideal case where all the leakages are neglected. Therefore,

(Equation S17) $$\begin{array}{l} L_{sr}^{2}\;=\;L_{ms}\;\times \;L_{mr} \end{array}$$

Substituting Equation S17 in S16

(Equation S18) $$\begin{array}{l} R_{r}^{\prime }\;=\;R_{r}\;\times \;\frac{L_{ms}}{L_{mr}}\; \end{array}$$

(Equation S19) $$\begin{array}{l} \Lambda_{r}^{\prime }=\;\frac{L_{ms}}{L_{rs}}\times \Lambda_{r} \end{array}$$

Equation S19 can further be modified by using the previously introduced parameters to get Equation S20

(Equation S20) $$\begin{array}{l} \Lambda_{r}^{\prime }=\;\frac{L_{ms}}{L_{rs}}\times \frac{Lms}{Lsr}\;I_{r}^{\prime }*Lr \end{array}$$

Similar to resistance in Equations S18, S20 can be reduced to Equation S21

(Equation S21) $$\begin{array}{l} \Lambda_{r}^{\prime }=\;\frac{L_{ms}}{L_{mr}}\;Ir^{\prime }*Lr \end{array}$$

$L_{r}$ and $L_{mr}$ represents the same quantity as there is no leakage. Therefore, Equation S21 can be written as Equation S22

(Equation S22) $$\begin{array}{l} \Lambda_{r}^{\prime }=\;Lms\;Ir^{\prime } \end{array}$$

Hence the primed rotor inductance can be written as shown in Equation S23

$$L_{r}^{\prime }=\;L_{ms}$$

#### Analysis of Kirchoff's law

The equivalent circuit shown in Figure S1 and 2E can be solved using Kirchoff’s law. For loop 1, the equation can be written as shown in Equation S23

(Equation S23) $$\begin{array}{l} V-\;I_{1}\;\times \;R_{s}-\left(I_{1}-\;I_{2}\right)\times Z_{m}\;=0 \end{array}$$

Similarly, the equation for Loop 2 can be written as shown in Equation S24

(Equation S24) $$\begin{array}{l} -\;\left(I_{2}-I_{1}\right)\;\times Z_{m}-\;I_{2}\times \frac{R_{r}}{s}\;=0 \end{array}$$

Adding Equations S23 and S24

(Equation S25) $$\begin{array}{l} V-\;I_{1}\times \;R_{s}-\;I_{2}\times \;\frac{R_{r}}{s}=0 \end{array}$$

Rearranging Equation S25 and solving for $I_{1}$

(Equation S26) $$\begin{array}{l} I_{1}=\frac{\left(V-\;I_{2}\times \;\frac{R_{r}}{s}\;\right)}{R_{s}}\; \end{array}$$

Substituting Equations S26 in S23

(Equation S27) $$\begin{array}{l} V-\left(\left(\frac{\;V-\;I_{2}\times \frac{R_{r}}{s}}{R_{s}}\right)\times \;R_{s}\right)-\left(\;\frac{\left(\;V-\;I_{2}\times \frac{R_{r}}{s}\right)}{R_{s}}-I_{2}\right)\times Z_{m}\;=\;0 \end{array}$$

(Equation S28) $$\begin{array}{l} I_{2}\;\times \;\frac{R_{r}}{s}-\frac{Zm}{Rs}\;\times \left(V-\;I_{2}\;\times \frac{R_{r}}{s}\right)\;+\;I_{2}\;\times \;Z_{m}=0 \end{array}$$

(Equation S29) $$\begin{array}{l} I_{2}\;\times \;\frac{R_{r}}{s}+\;I_{2}\;\times \;\frac{R_{r}}{s}\times \frac{Z_{m}}{R_{s}}\;+\;I_{2}\times Z_{m}=Z_{m}\;\times \frac{V}{R_{s}} \end{array}$$

Solving for $I_{2}$

(Equation S30) $$\begin{array}{l} I_{2}=\;\frac{Z_{m}\times V}{R_{s}\times \left(\frac{R_{r}}{s}+\;\frac{R_{r}}{s}\times \frac{Z_{m}}{R_{s}}+\;Z_{m}\right)}\; \end{array}$$

where

(Equation S31) $$\begin{array}{l} Z_{m}=j*\omega *\;L_{s} \end{array}$$

#### Power calculations

As per Figures S1 and 2E, power transmitted from stator field to the rotor can be written as shown in Equation S32

(Equation S32) $$\begin{array}{l} P_{in}=V\;I_{2} \end{array}$$

where

(Equation S33) $$\begin{array}{l} V=I_{2}*\frac{Rr^{\prime }}{s} \end{array}$$

Therefore,

(Equation S34) $$\begin{array}{l} P_{in}=\;\left|I_{2}\right|{\;}^{2}\;\frac{R_{r}^{\prime }}{s}\; \end{array}$$

For a three-phase supply, Equation S34 can be multiplied by a factor of 3. And hence the total power transmitted to the rotor in the proposed model is calculated using Equation S35

(Equation S35) $$\begin{array}{l} P_{in}=\;3\left|I_{2}\right|{\;}^{2}\;\frac{R_{r}^{\prime }}{s} \end{array}$$

#### Average magnetic field calculation

The magnetic flux (∅)in the system is given by Equation S36.

(Equation S36) $$\begin{array}{l} \emptyset =\;\int B\;\cdot dA \end{array}$$

Where B is the magnetic field crossing the area dA.

FEM calculations performed using the software packages were used to obtain the values of the average fluxes passing through the coils of the single-phase for 10 different cases as discussed in Table S1. Area of the ferromagnetic mound on which the coils wind is 7.05 x 10^-5^ m^2^.Therefore, the average value of the magnetic field is obtained by dividing the flux values obtained using FEM with the designated area. The values of flux and corresponding magnetic fields are summarized in Table S2.

#### Per phase stator inductance calculations

(Equation S37) $$\begin{array}{l} \Lambda =N\;\emptyset \; \end{array}$$

The flux linkage (Λ) can be calculated using Equation S37.

Substituting Equation S36 in S37 to obtain Equation S38

(Equation S38) $$\begin{array}{l} \Lambda =N\int B\;\cdot dA\; \end{array}$$

The inductance can therefore be calculated using Equation S39 by substituting it back in Equation S37. This is summarized in Equation S40.

(Equation S39) $$\begin{array}{l} \Lambda =L\;I_{0} \end{array}$$

(Equation S40) $$\begin{array}{l} L=\;\frac{1}{I_{0}}\;\int B\;\cdot dA\; \end{array}$$

## Footnotes

Supplemental information can be found online at https://doi.org/10.1016/j.isci.2021.102644.

## References

[bib1] Bartlett M.D., Markvicka E.J., Majidi C. (2016). Rapid fabrication of soft, multilayered electronics for wearable biomonitoring. Adv. Funct. Mater..

[bib2] Bernstein I.B., Fanucci J.B., Fishbeck K.H., Jarem J., Korman N.I., Kulsrud R.M., Lessen M., Ness N. (1961). An electrodeless MHD generator. Second Symposium on the Engineering Aspects of Magnetohydrodynamics.

[bib3] Caro F., Sadr R. (2019). The Internet of Things (IoT) in retail: bridging supply and demand. Bus. Horiz..

[bib4] Fan F.R., Tian Z.Q., Lin Wang Z. (2012). Flexible triboelectric generator. Nano Energy.

[bib5] Ghose A., Han S.P. (2014). Estimating demand for mobile applications in the new economy. Manage. Sci..

[bib6] Hsu T.-H., Manakasettharn S., Taylor J.A., Krupenkin T. (2015). Bubbler: a novel ultra-high power density energy harvesting method based on reverse electrowetting. Sci. Rep..

[bib7] Intani P., Sasaki T., Kikuchi T., Harada N. (2010). Analysis of disk AC MHD generator performance by finite element method. J. Plasma Fusion.

[bib8] Jackson W.D., Pierson E.S. (1964). Design Generators, considerations for MHD induction. Proc. Internat’l Symp. on Magnetohydrodynamic Electrical Power Generation.

[bib9] Kang S., Baek H., Jung E., Hwang H., Yoo S. (2019). Survey on the demand for adoption of Internet of Things (IoT)-based services in hospitals: investigation of nurses’ perception in a tertiary university hospital. Appl. Nurs. Res..

[bib10] Lipo T.A. (2017). Introduction to AC Machine Design.

[bib20] Woodson H. (1964). Magnetohydrodynamic a-c power generation. Proceedings of the 1962 Pacific Energy Conversion Conference.

[bib11] Miner, C.S., Chan, D.M., and Campbell, C. (2001). Digital jewelry: wearable technology for everyday life. In Conference on Human Factors in Computing Systems - Proceedings, (New York, New York, USA: ACM Press), pp. 45–46.

[bib12] Muralt P., Marzencki M., Belgacem B., Calame F., Basrour S. (2009). Vibration energy harvesting with PZT micro device. Procedia Chemistry.

[bib13] Panchadar K., West D., Taylor J.A., Krupenkin T. (2019). Mechanical energy harvesting using a liquid metal vortex magnetohydrodynamic generator. Appl. Phys. Lett..

[bib14] Roundy S., Wright P.K., Rabaey J. (2003). A study of low level vibrations as a power source for wireless sensor nodes. Comput. Commun..

[bib15] Strohl G.R., Jackson W.D. (2021). Magnetohydrodynamic power generator | physics. Encycl. Br.

[bib16] Wang T.C., Dudzinsky S.J. (1967). Theoretical and experimental study of a liquid metal MHD induction generator. AIAA J..

[bib17] Wang T.C., Dudzinsky S.J. (1967). Comparison of MHD induction generator analyses. AIAA J..

[bib18] West D., Taylor J.A., Krupenkin T. (2020). Alternating current liquid metal vortex magnetohydrodynamic generator. Energy Convers. Manag..

[bib19] Widdicks, K., Bates, O., Hazas, M., Friday, A., and Beresford, A.R. (2017). Demand around the clock: time use and data demand of mobile devices in everyday life. In Conference on Human Factors in Computing Systems - Proceedings, (New York, NY: Association for Computing Machinery), pp. 5361–5372.

