# Supplementary material for: Three-phase alternating current liquid metal vortex magnetohydrodynamic generator
Source: iScience. 2021 May 24;24(6):102644. doi: 10.1016/j.isci.2021.102644 (PMC8192567; doi:10.1016/j.isci.2021.102644)
Supplement: Document S1. Figures S1–S3 and Tables S1–S3 [file mmc1.pdf]

iScience, Volume *24*

Supplemental information

Three-phase alternating current liquid metal vortex magnetohydrodynamic generator

Siddharth Raj Gupta, J. Ashley Taylor, and Tom Krupenkin

Supplemental Information

Supplemental data items

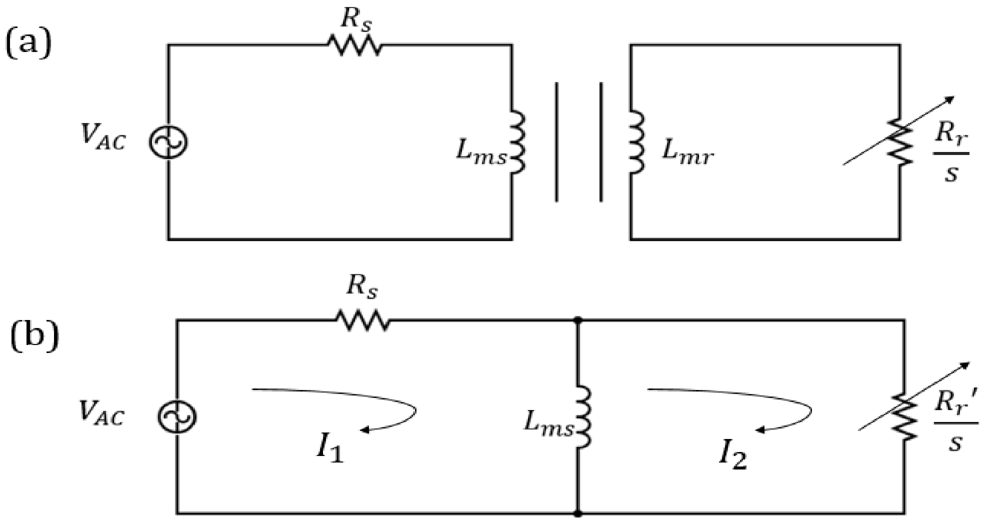

Figure S1. Equivalent circuit (Related to Figure 2(d) and 2(e))

(a) Per-phase exact equivalent circuit

(b) Per-phase approximated equivalent circuit

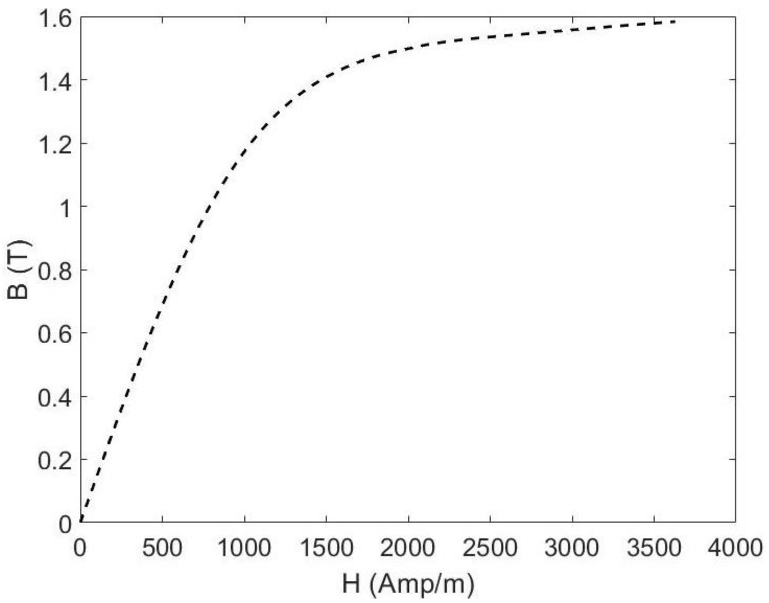

Figure S2. BH curve (Related to Table 1)

Device application

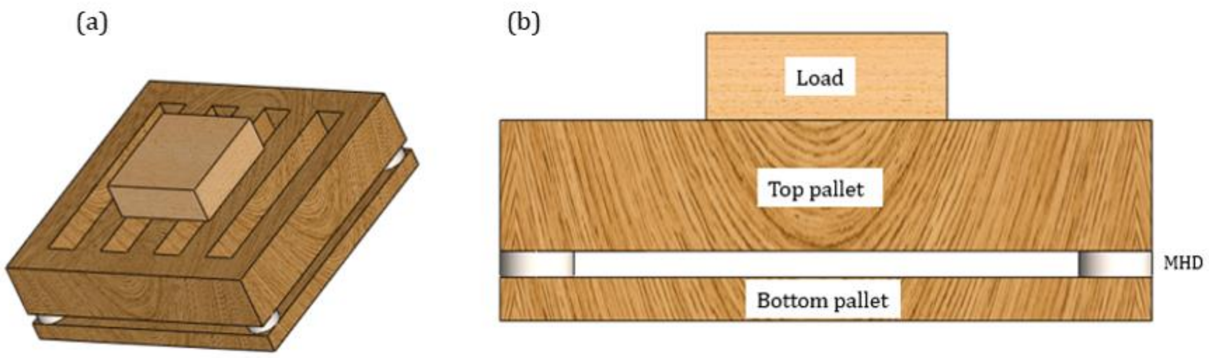

Figure S3. Shipping container pallet assembly fitted with MHD at corners (Related to Figure 1)

a) 3D view
b) Side view

One of the area where this device can be used is high force (between 1000N-10000N) and low displacement (between 1mm – 20mm) operations. A possible application is in transportation using pallets of shipping containers. Our proposed device can be positioned at all the four corners of a shipping pallet. The weight of the load and small vibrations together can act as a source to provide the fluid flow in the MHD. A battery will be required as a starter but the continuous motion of the container will result in continuous power output from the MHD. This can therefore be used for various Internet of Things (IoT) applications. One of the important applications can be GPS positioning. The MHD can power the positioning sensors and can transmit the real time location of the shipment. Further, the output power can be used to power up temperature sensors which can keep a record of the container's temperature throughout the journey and can help in transport of various temperature sensitive medicines and food products. Since the device is capable of producing continuous power it can also be utilized to recharge the battery keeping the sensors running for the entire journey.

Table S1. Magnetic field at respective supplied current (Also see Table 1)

**Current (A)**	0.1	0.2	0.3	0.4	0.7	1.1	1.3	1.6	2	3	4	5	6
**B (T)**	0.23	0.43	0.58	0.70	0.80	0.85	0.87	0.89	0.93	0.99	1.05	1.10	1.14

Table S2. FEM data (Related to Figure 3)

**Number of turns**	**Applied current (A)**	**Average flux (Wb)**	**Flux linkage (Wb-turns)**	**Average magnetic field (T)**	**Inductance (H)**
50	0.1	1.67E-05	8.33E-04	0.20	8.33E-03
	0.2	3.11E-05	1.56E-03	0.43	7.78E-03
	0.3	4.22E-05	2.11E-03	0.58	7.03E-03
	0.4	5.09E-05	2.55E-03	0.70	6.36E-03
	0.7	5.77E-05	2.89E-03	0.80	4.12E-03
	1.1	6.18E-05	3.09E-03	0.85	2.81E-03
	1.3	6.31E-05	3.15E-03	0.87	2.43E-03
	1.6	6.48E-05	3.24E-03	0.89	2.03E-03
	2	6.71E-05	3.36E-03	0.93	1.68E-03
	3	7.18E-05	3.59E-03	0.99	1.20E-03
	4	7.57E-05	3.79E-03	1.05	9.47E-04
	5	7.93E-05	3.97E-03	1.1	7.93E-04
	6	8.24E-05	4.12E-03	1.14	6.87E-04

Table S3. Parameter Table (Related to Figure 2(d) and 2(e))

**Parameter**	**Value**
$\boldsymbol{L_{ms}}$	0.014 H
$\boldsymbol{R_s}$	0.22 Ω
$\boldsymbol{R_r}$	0.016 Ω
